# The key actors maintaining elders in functional autonomy in Bobo-Dioulasso (Burkina Faso)

**DOI:** 10.1186/1471-2458-14-689

**Published:** 2014-07-05

**Authors:** Abdramane Berthé, Lalla Berthé-Sanou, Serge Somda, Blahima Konaté, Hervé Hien, Fatoumata Tou, Maxime Drabo, Fatoumata Badini-Kinda, Jean Macq

**Affiliations:** 1 Muraz Centre, 01 BP 390 Bobo-Dioulasso 01, Burkina Faso; 2 Institut de Recherche Santé et Société, Université Catholique de Louvain (UCL), Bruxelles, Belgium; 3 Burkinabe Public Health Association (ABSP), Ouagadougou, Burkina Faso; 4 Health Sciences Research Institute (IRSS), Ouagadougou, Burkina Faso; 5 National Public Health Laboratory (LNSP), Ouagadougou, Burkina Faso; 6 Sociology department, Ouagadougou University, Ouagadougou, Burkina Faso

**Keywords:** Aged, Disability evaluation, Disabled persons, Old age assistance, Social support, Africa south of the Sahara

## Abstract

**Background:**

Globally, a significant increase in functional disability among the elderly is expected in the near future. It is therefore vital to begin considering how Sub-Saharan Africa countries can best start building or strengthening the care and support system for that target population. Study objectives are: 1) identify the key actors of the social system who maintain elders in functional autonomy at home in Bobo-Dioulasso (Burkina Faso) and 2) to describe the functional status of older people living at home.

**Methods:**

We conducted a longitudinal descriptive study among the elderly aged 60 and above (351). Their functional status was evaluated using the Functional Autonomy Measurement System (SMAF). Data analysis was done using the statistical software package STATA (SE11).

**Results:**

In Bobo-Dioulasso, 68% of seniors have good functional capacity or a slight incapacity and 32% have moderate to severe incapacities. Older people die before (3%) or during (14%) moderate to severe disabilities. This would mean that the quality of medical and/or social care is not good for maintaining functional autonomy of older people with moderate to severe disabilities. Two main groups of people contribute to maintain elders in functional autonomy: the elderly themselves and their family. Community, private or public structures for maintaining elders in functional autonomy are non-existent. The social system for maintaining elders in functional autonomy is incomplete and failing. In case of functional handicap at home, the elders die. But stakeholders are not conscious of this situation; they believe that this system is good for maintaining elders in functional autonomy.

**Conclusion:**

It is likely that the absence of formal care and support structure likely shortens the lifespan of severely disabled older people. Stakeholders have not yet looked at this possibility. The stakeholders should seriously think about: 1) how to establish the third level of actors who can fulfill the needs to maintain elders in functional autonomy that are not satisfied by others (family members or the older individuals themselves), and 2) how to reinforce the role of each actor and the collaboration between the different groups of people of this system.

## Background

Older population is increasing faster in sub-Saharan Africa than anywhere else [1]. This demographic transition will be followed by an epidemiological transition, with an increase of chronic diseases and of associated functional disability, especially among older people. Given these changes, new strategies will be needed to provide adequate health services and support for older people [2,3]. A major aspect that will need to be addressed is the management of functional disability. In order to develop appropriate strategies, it is imperative to identify people suffering from functional disability [4], to assess the nature and extent of these disabilities [5] and to characterize the existing social support systems that help people to cope with them.

There is a growing interest in research about the global well-being of older people and/or their disabilities within sub-Saharan Africa. Few data exist regarding their functional status. Studies conducted to date [3,6-13] report a prevalence of disability in older people that varied between 2% and 54%, depending on the country (Botswana, Burkina Faso, Mali, Nigeria, Zimbabwe, etc.) and time (from 1999 to present). Differences in the operational definitions, the targeted disability domains, the population’s profile, the data collection tools, the context and even the threshold or degree of disability measured, make difficult to compare or project estimates of older peoples’ needs for help across different settings [11-14].

We report here the results of a study conducted in Bobo-Dioulasso, Burkina Faso. It was a first step of a larger study. This latter aimed to exploring how the Burkinabe society, and more specifically how the family support system is organized to maintain older people with functional disability at home. Results were reported elsewhere [4,5,15].

The sub-study presented here aimed at responding to the following research questions: 1) who are the key actors of the social system who maintain elders in functional autonomy at home? And 2) what is the functional status elders living at home in Bobo-Dioulasso (Burkina Faso). In the questionnary SMAF, these 2 questions correspond respectively to the second column (resources) and to the first column (incapacity). So these study objectives are: 1) identify the key actors of the social system who maintain elders in functional autonomy at home and 2) to describe the functional status of older people living at home.

## Methods

We conducted a longitudinal study (February - April 2011 and February - April 2012) among a sample of older people, aged 60 or older in Bobo-Dioulasso (Burkina Faso), who still reside at home. In 2006 [16], this town had 489,967 habitants, 18,130 (3.7%) of which are more than 60.

In order to study and evaluate functional disability in this population, we used PRISMA-7 (a screening instrument) and the SMAF Functional Autonomy Measurement System (Système De Mesure De L’Autonomie Fonctionnelle). Both instruments were applied to the whole sample. We report here only on the results of the SMAF (results of the combined use of PRISMA-7 and SMAF have been reported elsewhere [4]). The SMAF is copyrighted and cannot be used without permission from the Health Expertise Center in Sherbrooke (Canada). The development of this measurement tool was guided by the World Health Organization’s International Classification of Functioning, Disabilities and Handicaps (ICF). The SMAF evaluates functional disability among older people in 5 domains: activities of daily living (ADL, 7 items), mobility (MOB, 6 items), communication (COM, 3 items), mental functions (MF, 5 items), and instrumental activities of daily living (IADL, 8 items). It is therefore composed of 29 items/activities or tasks. For each item, the level of disability is given a score between 0 (independent or good functional ability) and 3 (entirely dependent on others for activities). The minimum SMAF score is 0, and the maximum is 87. An overall score of zero (0) signifies a good functioning ability; a score between 1 and 14.5 signifies a mild disability, without the need for assistance; a score greater than or equal to 15 indicates moderate to severe disabilities (or the need for supervision, partial or total assistance). In addition to functional disability, it measures (for each item) the availability of resources to compensate the disability, the type of resources, resource stability, handicap (i.e. unmet needs or a disability that is not compensated by the use of sufficient resources), functional independence (a balance between disability and the use of resources to compensate the disability) [17-22]. SMAF facilitates the identification of the different actors who contribute to maintain elders in functional autonomy.

The following steps were taken to adapt the SMAF for use in the context of Bobo-Dioulasso. First, the instrument was reviewed by the members of the research team, all experts on the culture and context of Bobo-Dioulasso. The following items were modified to account for the local context: 1) “using a wheelchair indoors” was changed to “walking around with the help of a cane or a wheelchair in the concession 2) “using the telephone” was change to “using the telephone, radio or television”; 3) “budget management” was change to “management of budget or material goods”. The instrument was translated into Dioula (the local language). Secondly, two pre-tests were done in the periphery of Bobo-Dioulasso (outside the research study). The results of the pre-test were discussed by the research team. No major problems with the application of the SMAF in this context were identified during the pre-test. One of the main findings of the pre-test was that there was great variety in the living situations of the elderly in Bobo-Dioulasso (type of housing, persons who live with the elderly, etc.) One advantage of the SMAF is that it is flexible and can accommodate a variety of individual situations, regardless of culture, country or continent. In Bobo-Dioulasso, some older people have living situations similar to those found in Europe and while other live in conditions more commonly found in rural sub-Saharan Africa.

Bobo-Dioulasso is divided into 25 geographical sectors within three districts. The study was conducted in 18 sectors. The other seven sectors were excluded because they do not have formal street numbers. In this study, we used a systematic random sample. The sampling frame was comprised of people over 60 living in the 18 included sectors. The choice of compounds (groups of residences within a walled area, usually occupied by members of the same family) within each sector was done by a systematic drawing, using a step of 5 from the sector centre. The first compound in each sector was selected by spinning a bottle in the clockwise direction. If only one elder lived in the compound, he or she was asked to participate in an interview. If more than one elder lived in the compound, one was randomly selected. If the selected person was absent two more attempts were made to interview that person. If the person was still not at home at the time of third visit, he or she was replaced by another elder living in the compound.

We calculated a sample size of 377 interviewees from the older population in Bobo-Dioulasso (N = 18,130); with an expected prevalence of 50%. Accuracy or margin of error was fixed at 5%, and 95% for the degree of confidence. The number of interviewees in each of the three boroughs was proportional to the number of households in the inhabited, geographical sector.

Participation was free and voluntary. Informed consent was obtained from all participants. If a participant refused after having read the consent form, he or she was not replaced.

The research protocol was approved by the National Ethics Committee for Health Research in Burkina Faso.

One single interviewer (social worker) administered the SMAF to all of the participants in Dioula or French (according to the preference of the participant). He tested capacities of older persons in the communication domains (to listen, see, and speak) and for the mental function (to understand, judge, etc). For activities of daily life, domestic life or mobility, he completed SMAF, based on the declaration of older persons or their relatives.

The evaluator effectively assessed what people were doing and not only what they were able to do. For instance, when somebody was cooking for them, and even they would be able to cook, they would be considered as having incapacity. In that sense, we remained fidel to SMAF logic.

All of the questionnaires were completed and verified. Data entry was conducted using the CSPro 4 program, which allowed for masked data input. Data was then analyzed using Stata 11 SE. The results of this study were restituted to the population and the stakeholders.

## Results

Of the 377 older people who were contacted, 362 people provided informed consent and began the study; 351 an- swered all of the SMAF questions the first year (February - April 2011), 305 do it the second year (February - April 2012). The second year 57 elderly (28 absents, 22 dead and 7 refusal) did not answered.

### Socio-demographic characteristics of the participants

The majority of people in our sample (n = 351) were: between the age of 60 and 75 (80%), female (59%), illiterate (68%), married (50%), the owner of a house (84%), without a regular income (62%), and Muslim (72%). On average, these people had seven children, lived in a two-household structure (plot for residential use) with 10 other people and had lived in Bobo-Dioulasso for 47 years and in their geographical sector for 36 years.

### The elderly as first and principal actors responsible for maintaining their own functional autonomy in Bobo-Dioulasso

Among the participants in the study (351), the lowest SMAF score was zero (00) and the highest was 60. The average score was 14 (95% CI: 12.66 - 14.65). The median score was 12. Two-thirds (240/351 or 68%, 95% CI 63.23 – 73.21) of older people had good functional capacity or a slight incapacity (SMAF score ≤ 15). The average score was 9 (95% CI: 8.56 - 9.57) and the median score was 10.

The two-thirds of older people with SMAF scores ≤ 15 were primarily responsible for maintaining their own functional autonomy. They did not require supervision by or assistance from another person. They were autonomous in all five SMAF domains: ADL, MOB, COM, MF and IADL.

### The family as the second group of people for maintaining elders in functional autonomy in Bobo-Dioulasso

A third of the older people (111/351 or 32%; 95% CI: 26.79 – 36.77 showed moderate to severe disability and therefore a need for supervision or assistance from another person (SMAF score ≥ 15). In this third (n = 111), the average SMAF score was 23 (95% CI: 21.62 - 25.5) and the median score was 20.

Only one person showed severe disability. This was 0.3% of all the elderly (1/351, 95% CI: 00.01 – 01.58) and 1% of older people in moderate to severe disability (1/111, 95% CI: 00.02 – 04.92). His SMAF score was 60.

**Figure 1:**
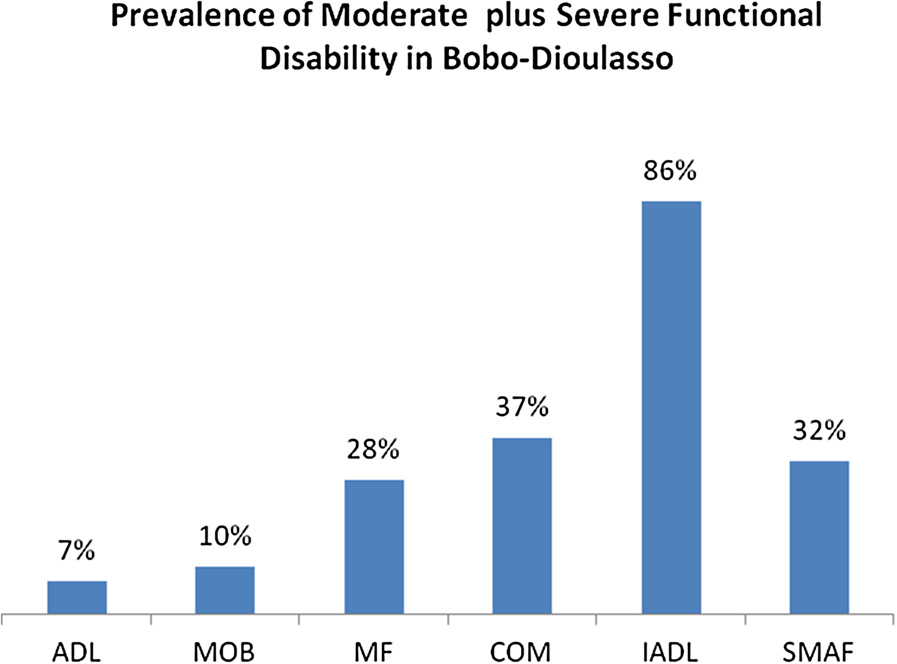
Variation in prevalence of moderate or severe functional among older people in Bobo-Dioulasso.

Persons with moderate to severe functional disability were functionally disabled in either two (2%), three (14%), four (46%) or five (38%) SMAF domains.The prevalence of moderate to severe multidimensional functional disability (32%), varied according to the different SMAF dimensions/domains: it ranges from 7% (ADL) to 86% (IADL), as illustrated in Figure 1.

In each domain, the prevalence of moderate to severe functional disability, and thus the need for supervision or assistance, varies across activities/items within a specific domain, as illustrated by Table 1. The 29 activities or items in this table can be regrouped into three broader groups:

**Table 1:** Need for supervision, partial or complete assistance according to activities (n = 351)

**Activities of daily living 7%**	Washing 67%	Maintaining bladder function 23%	Going to the toilet 8%	Personal maintenance 4%	Getting dressed 4%	Maintaining bowel function 4%	Eating 2%	
([04.66 – 10.33] CI 95%)								
**Mobility 10%**	Walking outdoors 11%	Using a cane 4%	Transportation 4%	Walking indoors 2%	Using the stairs 2%	Using a prosthesis 0%		
([07.04 – 13.59] CI 95%)								
**Mental function 28%**	Memory 23%	Behaviour 16%	Making judgments 12%	Orientation 11%	Understanding 6%			
([23.56 – 33.23] CI 95%)								
**Communication 37%**	Seeing 34%	Hearing 8%	Speaking 2%					
([32.25 – 42.61] CI 95%)								
**Instrumental activities of daily living 86%**	Preparing meals 82%	Doing laundry 80%	Household upkeep 66%	Using the telephone, television or radio 71%	Using public transportation 32%	Going shopping 21%	Taking medications 5%	Budget management 4%
([81.65 – 89.24] CI 95%)								

• The first group is made up of activities for which fewer than 10% of people required supervision or assistance (15/29 items). These activities include: using a prosthesis, the use of the terrace, walking indoors, speaking, eating, maintaining bowel function, getting dressed, personal maintenance, using a cane, transportation, budget management, taking medication, understanding, listening, using the toilet;

• The second group consists of nine activities that required assistance or supervision for between 11% and 34% of people. These activities include: walking outdoors, orientation, making judgments, behavior, maintaining bladder function, shopping, memory, using public transportation, and seeing;

• The third group is comprised of five activities for which between 66% and 82% of people needed supervision or assistance (5/29 items). These activities include: household upkeep, washing, using the telephone/television/radio, doing laundry, and preparing meals (these are instrumental activities).

All the elderly with a moderate to severe functional disability (111/351 or 32%) who expressed a need for assistance in a certain domain reported having support systems available to satisfy the specific needs. These sources of support were stable (i.e. they were not expected to increase or decrease in the 2 to 3 weeks following the survey) in almost all cases. These social support systems for maintaining functional autonomy were almost exclusively made up of family members. In some rare cases, they included caretakers, neighbors or friends. With these resources available, the score or level of handicap (defined as a disability that is not compensated by resources) among elderly people is therefore zero. In other words, in our study’s sample, not one elder with a functional disability is lacking in human resources to help manage their disability; their needs are almost exclusively met by family members. So, the family members constitute the second group of actors who maintain elders in functional autonomy in Bobo-Dioulasso.

In the second year of the study, 6% (22/362) of the elderly had died. This proportion was 3% (7/251) among those who were without disabilities or with slight disabilities and 14% (15/111) among those who were in moderate to severe disabilities. Among those who were without disabilities or with slight disabilities, 19% went into moderate to severe disabilities.

### Community, private or public structures for maintaining elders in functional autonomy are non-existent

**Figure 2:**
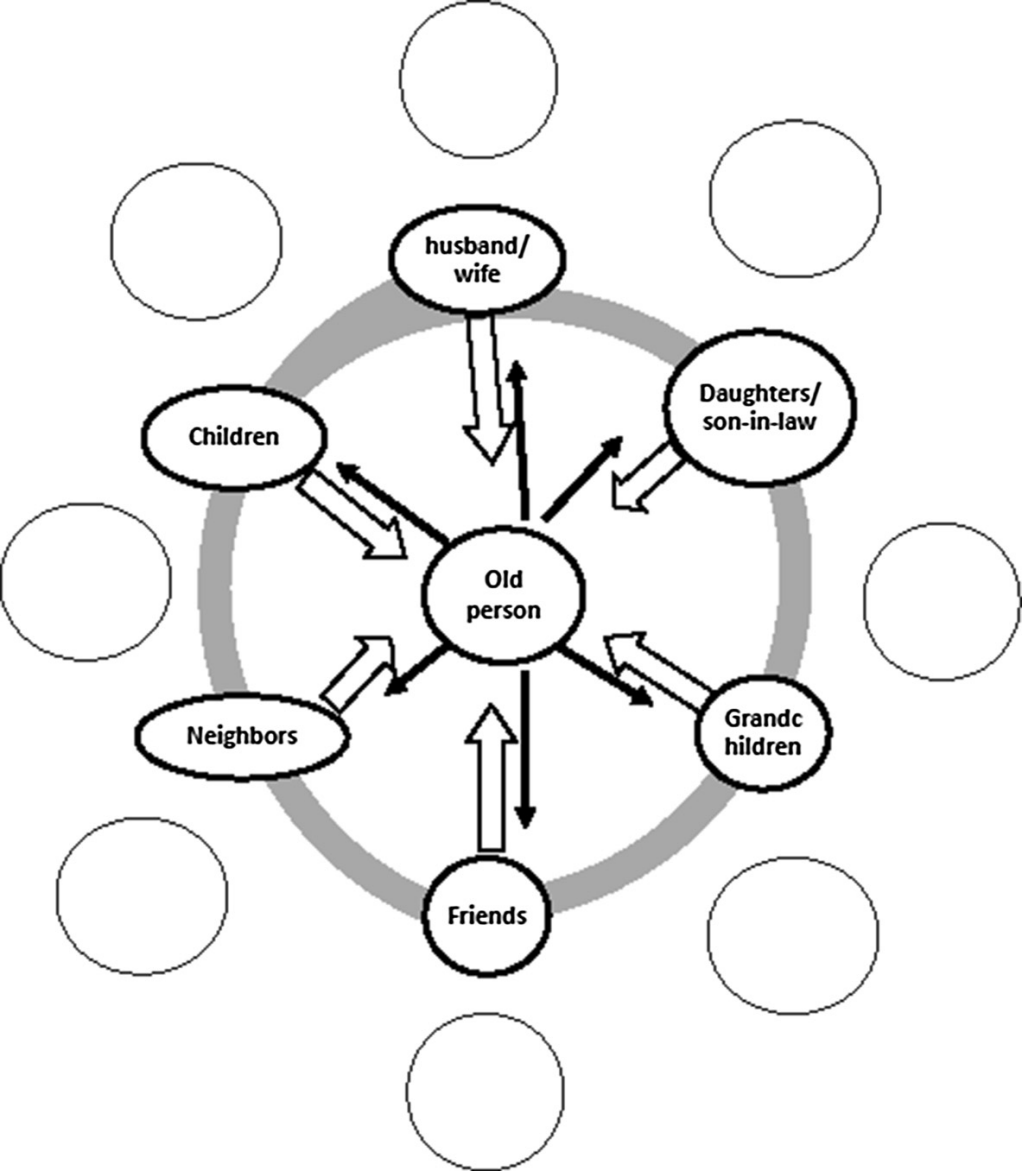
Family around the older person for maintaining his functional autonomy and no formal or informal structure to support the family.

Not one person among the elderly with moderate to severe disabilities (111/351 or 32%) cited health workers, volunteers in associations and/or other agents of community, private or public structures as actors who maintain elders in functional autonomy. In Bobo-Dioulasso these types of services do not exist, formally or informally (see Figure 2). Social support for maintaining elders in functional autonomy is thus provided entirely at the individual and household/family levels. There is a complete lack of community, organizational or government resources to support individuals and their families and thus lessen their burden.

## Discussion

This study is one of the few conducted in a French speaking West African context that provides a comprehensive description of the functional status of older people, and characterizes of the different groups of people contribute to maintain elders in functional autonomy.

### Possible explanations for the levels of disability in older people of Bobo-Dioulasso

Older people from Bobo-Dioulasso have an average SMAF score of 14, which corresponds to a mild disability. Those with a moderate to severe disability have an average SMAF score of 23. Only one person in this survey met the criteria for classification as severely disabled. There are two possible explanations for these results. Firstly, given that capacities assessment was based on declaration for some of the function, it is possible, but unlikely, that older people would have underestimated their level of disability.

Secondly, the most likely explanation is that older people in this setting die before they reach a state of severe disability, or soon after becoming severely disabled. Mortality was near five times higher in elderly with moderate to severe disabilities than those without disabilities or with slight disabilities (14% versus 3%). Moreover, in one year, 19% (40/215) went into moderate to severe disabilities, it is possible that the 6% of deaths among people without disabilities or with a slight disabilities went quickly into moderate to severe disabilities which have been very fatal to them. This could be an indication that the quality of care and support available is not sufficient [23] for maintaining the functional autonomy of the elders with moderate to severe disabilities in their homes. In this context, to live with a moderate disability is to beat death’s door and to develop severe disability is to be essentially dead. In this society, moderate to severe functional disabilities are often considered normal diseases of old age, that inevitably lead to decline and death and about which we can do nothing.

A relatively low prevalence of moderate to severe disability has also been reported in other African contexts. According to the 2006 census in Burkina Faso, 7% of older citizens have a major handicap [7]. The estimated prevalence of multidimensional, moderate to severe/functional disability among people aged 60 and older is 43% in low-income countries, according to the 2011 World Report on Disability [12] and is 54% in Africa according to a WHO report on the global burden of morbidity [12]. The prevalence of moderate to severe disability in Africa varies spatially and temporally [3,6,9,10,13].

All older people with a moderate to severe functional disability (32%) have a disability in at least two SMAF domains. Functional disability is all-inclusive; in some sense it is a “communicating vessel” suggesting a multitude of dimensions. Disabilities in one domain, such as MOB, MF or COM, can easily have an effect on ADL or IADL.

This study is one of the few studies that describe functional disability among the aging population in Burkina Faso. The results can be extrapolated to the general older population with the socio-demographic characteristics of the older people from Bobo-Dioulasso. However, the socio-cultural specificities of Bobo-Dioulasso (a predominantly Muslim city, and a geographical setting that favors intergenerational cohabitation) must be considered when making generalizations from the results of this study. Moreover, the proportion of people with a handicap (0%) found in this study does not deny the existence of elders without human resources (parents, friends, neighbors) in Bobo-Dioulasso, but only emphasizes the rarity. The number of older people living alone is also very low (3%) in this area [7].

### Lessons learned by identifying the actors of the social system who maintain elders in functional disabilities

In Bobo-Dioulasso, the first and principal actors who maintain older people in functional autonomy are older people themselves. They are 68% of older people in this case. The second set of actors responsible for the maintenance of elders in functional autonomy is family members, friends and neighbors. A third of older people (32%) or all older people with moderate to severe disabilities receive some supervision and/or partial or total assistance. The resource is stable.

Studies of functional disability in Africa south of the Sahara [3,6,9,10,12,13] have shown that family members are the primary group that maintains elders in functional autonomy. According to Ouakam-Ouakam [9], a study conducted in Bamako (Mali) in 2004 found that 81% of seniors living at home with or without functional disabilities reported that they would not able to live without supervision, and/or partial or complete assistance from their family. This has led some researchers to measure the availability of family members or the family index [24], the ratio of family support to elders [25], the dependency ratio [26], which estimates the number of adults available for the elders or the type of people who live with the elderly (in terms of their relationship to the elders). In the state of Imo in Nigeria, for example, according to Unanka [25], the number of people under 60 that are available to help older people, divided by the total number of older people in the same household goes from zero (0) to eight (8), with an average of three (3) people per elder in each household. In 18% of households there were zero available people per elder. This study therefore measured the availability of family members to help an older member of their family.

One must note that there is often a gap between what is said and what really exists in terms of support. The context of the Nigerian study mentioned above is very different from the one in Bobo-Dioulasso. Within the Bobo-Dioulasso setting/study, there were no elder with a functional disability, who required but did not receive support and social care (older people with a handicap) and thus there were no people who were forced to resort to formal networks to maintain independence. This could be explained by the social and cultural norms regarding older people in Bobo-Dioulasso (as described above) [27]. Additionally, the sample was restricted to people living in compounds/houses (often with other family members in co-residence) and did not include homeless older people or those who spend their days begging in city centers (people who are often ostracized and/or treated as ”witches”).

The SMAF evaluates availability, type and stability of social resources; it does not look at quantity, quality or the reserves of these resources. The different transitions (demographical, epidemiological, socio-cultural, political and economical) [2,6,28,29] that the country is going through will bring about a decrease in the quality and quantity of these resources, and an increase in the number of the elderly with a functional disability, or even handicap. People within this region will be old before becoming rich [30,31].Figure 2 highlights the absence of formal services and organizations that provide support for maintaining elders in functional autonomy. It is possible that the strong presence/availability of social resources that provide an informal version of social care to the aged with a functional disability could explain the absence or weakness of the infrastructure of official care provided by the state, private organizations and faith-based communities. On the other hand, perhaps it is the absence of formal structures that led to the development of strong, social, and informal resources. Likely there is a degree of circular causality, where the development or under-development of one type of resource (informal or formal respectively) is both a cause and effect of the under-development or development of the other.

In this city where there are no formal services and organizations to maintain elders in functional autonomy, it is reasonable and logic that those who have moderate to severe functional disabilities in one or more areas of SMAF, and who completely lack support from family members, neighbors and friends, will not be able to survive and will die prematurely (compared to those who do have access to such informal social support systems) because we are in the functional area. In Bobo-Dioulasso, few actors (e.g. researchers, practitioners, leaders of social organizations and policy makers) are conscious of this situation. During the restitution, stakeholders have publicly declared that in Bobo-Dioulasso (or in Burkina Faso more broadly), there is not yet a need for nursing homes or assisted living facilities (homes for maintaining elders in functional autonomy). This is another potential example of circular causality: stakeholders do not perceive a need for formal support systems for maintaining elders in functional autonomy because the population of people who are completely functionally handicapped is extremely low; however, this may be due to high mortality rates within this population precisely because formal support services are unavailable. Thus the nil rate of functional handicap cannot necessarily be viewed as an indicator of the strength of social organizations providing support for the elderly. Elsewhere in sub-Saharan Africa [32-34], the stakeholders, the caregivers and even the general population also resist the suggestion that there is a need for nursing home or assisted-living facilities for the elderly, but everyone agrees the strengthening of formal community support to help the elders. Formal (community, confessional, private or public) structures are indispensable to complete sub-Saharan Africa social system for maintain elders in functional autonomy. If not, many elders will continue to die when families are failing.

### Study limits

The socio-cultural specificity of Bobo-Dioulasso (majority of Muslims, geographical space favorable to intergenerational cohabitation) must be taken into account in the results interpretation. The proportion of older people living alone is indeed very low in our study. This explains why no older people without assistance to help them has been found in this study.

Also, as already mentioned before, it is possible that scores have been under-evaluated for some items assessed only through older person or relative’s declaration. However, scores for these items remain higher than those found in other studies from sub-Saharan Africa.

## Conclusions

The health of older people is intimately related to their functional status. Our study showed a moderate to severe functional disability, with a need for supervision, or a need for assistance (32%) that was relatively high, compared to previous studies conducted in Africa. The extent of reported disability across domain or items was highly variable. The socio-cultural context in Bobo-Dioulasso, where generational and/or gender divisions of domestic labor persist, may explain the high local prevalence of moderate to severe functional disabilities and low prevalence of severe disabilities compared to other studies.

Two main groups contribute to maintain elders in functional autonomy: the elderly themselves (68% of older people) and their family including friends, neighbors (32% of older people or 100% of older people with moderate to severe functional disabilities). Community, confessional, private or public structures for maintaining elders in functional autonomy are non-existent. In case of functional handicap at home, it is evident that the elders die. But, in Bobo-Dioulasso and/or in Burkina Faso, stakeholders are insufficiently aware of this situation.

We argue that, in Burkina Faso and similar contexts, in order to provide adequate resources for maintaining elders in functional autonomy, there is a need for a tripartite social system comprised of three levels: 1) the elderly themselves, 2) the family and 3) the formal or informal institutions and organizations that target elders (associations, and/or confessional, private and/or public structures). The stakeholders in Bobo-Dioulasso and/or in Burkina Faso should seriously think about: 1) how to establish the third level of social support and resources for maintaining elders in functional autonomy in order to fill the gaps left by the two other levels; and 2) how to reinforce the role of each actor and strengthen collaborations between the three levels of the social support system.

## Abbreviations

SMAF: Functional autonomy measurement system (or Système de Mesure de l’Autonomie Fonctionnelle); ADL: Activities of daily life; MOB: Mobility; COM: Communication; MF: Mental function; IADL: Instrumental activities of daily life.

## Competing interests

The authors declare: no support from any organization for the submitted work; no financial relationships with any organizations that might have an interest in the submitted work in the previous three years, no other relationships or activities that could appear to have influenced the submitted work. The authors declare that they have no competing interests.

## Authors’ contributions

All authors contributed substantively in the development of this study (design, data composition, reviewing analysis and interpretations). All authors edited the final paper and thus the findings, interpretations, and conclusions in this paper represent their views. AB and JM act as guarantors. All authors read and approved the final manuscript.

## Authors’ information

All authors are investigators in the Inter-university Project Focusing on Older People in Burkina Faso (2011-2016). AB, LBS, BK, HH, FT, SS are PhD students. AB conducts research on family support for the elderly with functional disabilities and living at home. LBS has assessed the health program of the elderly in Burkina Faso (2008-2012). She specializes in program evaluation. BK is currently conducting a study on intergenerational family living, health promotion and health systems in Burkina Faso. HH conduct research on the role of the health care system and social support for appropriate management of medications among the elderly with co-morbidities in Burkina Faso. FT is doing a mapping of actors who identify seniors in Burkina Faso as their target population to build a network. SS is responsible for all statistical aspects of these related studies. These PhD students are supervised by MD, FBK and JM who are team leaders in their research institute. They have conducted various studies on the elderly. They look the epidemiological, public health (MD and JM) and socio-anthropological (FBK) aspects. All authors are active members or honorary members of the Burkinabe Public Health Association (ABSP).

Abdramane BERTHE holds a MA in Social Sciences, PhD in Public Health at the Catholic University of Louvain (UCL), Belgium. He is a researcher at the Centre Muraz (Burkina Faso Ministry of Health Institute of Training and Biomedical Research).

## Pre-publication history

The pre-publication history for this paper can be accessed here:

http://www.biomedcentral.com/1471-2458/14/689/prepub
